# Back to Basics: A Curriculum to Address the Pediatric Cardiac Anesthesia Workforce Crisis

**DOI:** 10.1111/pan.70074

**Published:** 2025-11-04

**Authors:** Lindsey Loveland, Susan C. Nicolson

**Affiliations:** ^1^ The University of Pennsylvania Perelman School of Medicine Philadelphia Pennsylvania USA; ^2^ Children's Hospital of Philadelphia Philadelphia Pennsylvania USA

## Abstract

The field of pediatric cardiac anesthesia faces a critical workforce shortage. Survival of children with congenital heart disease (CHD) has improved dramatically, increasing both lifetime procedural demand and case complexity. At the same time, the supply of fellowship‐trained pediatric cardiac anesthesiologists is shrinking due to an aging workforce, declining fellowship recruitment, financial disincentives, and concerns about work–life balance and professional culture. The resulting mismatch between demand and supply threatens access to care and risks moral injury and attrition within the specialty. At our institution, workforce pressures necessitated the redistribution of some CHD care to general pediatric anesthesiologists through the Special Cardiac Care Anesthesia (SCCA) model. As this team expanded, members themselves recognized a pressing need for structured education in congenital cardiac physiology and anesthetic implications. In response, the *Cardiac Basics* curriculum was developed to provide accessible, foundational cardiac content for anesthesiologists, fellows, advanced practice providers, and intensivists. The program was designed with three goals: (1) to provide essential cardiac knowledge, (2) to deliver material in a format accessible to diverse learners, and (3) to foster a psychologically safe environment where all participants could ask questions and engage openly. The curriculum uses weekly topic guides, brief interactive didactic sessions, and case‐based reinforcement. It has been well received, with strong participation across learner groups and overwhelmingly positive feedback highlighting clarity, brevity, and clinical applicability. Educational innovations such as *Cardiac Basics* represent a pragmatic and scalable strategy to address the pediatric cardiac anesthesia workforce crisis. By equipping a broader group of providers with the skills to care safely for selected CHD patients, such curricula can serve as a critical “force multiplier” while longer term solutions—fellowship recruitment, pipeline repair, and cultural change—are pursued.

## 1 Background

Pediatric cardiac anesthesia is facing a profound workforce crisis, particularly in the United States, where patient demand has outpaced the supply of trained specialists. Advances in the care of congenital heart disease (CHD) have led to improved survival across all ages, dramatically increasing both the lifetime volume and complexity of procedures requiring anesthesia [1]. Cases are longer due to redo sternotomies and multistage catheter‐based interventions, and new therapies such as hybrid ventricular assist device implantation and percutaneous valve procedures require anesthetic support outside the operating room [2, 3]. Delayed case starts due to limited intensive care unit capacity further extend clinical workloads, frequently into evenings and weekends.

Meanwhile, the supply of pediatric cardiac anesthesiologists is contracting. A recent US workforce survey revealed that nearly half of programs are actively recruiting, with projections of 120–194 additional anesthesiologists needed in the next 5–10 years [4]. More than half of the workforce is age 55 or older, and attrition unrelated to retirement or the COVID‐19 pandemic is significant, often related to moral injury—the distress of knowing what care is needed but being unable to provide it due to systemic constraints [5, 6].

Recruitment into pediatric anesthesia and pediatric cardiac anesthesia fellowships has also declined. In 2023, unmatched pediatric anesthesia fellowship positions rose from 17 in 2017 to 86, with more total positions than applicants across all ACGME‐accredited programs. Of 34 US pediatric cardiac anesthesia fellowship positions offered in 2023–2024, only 21 were filled [7]. Educational debt, minimal salary differentials, unpredictable hours, and negative perceptions of professional culture further discourage trainees [2, 3].

These combined pressures forced our institution to redistribute care for selected CHD patients to a subgroup of general pediatric anesthesiologists—the Special Cardiac Care Anesthesia (SCCA) team. Initially, the SCCA team was composed of dual‐trained practitioners in pediatric anesthesia and pediatric critical care medicine, who were already experienced in managing CHD patients in the pediatric intensive care unit. As procedural demand grew, however, this group alone could not meet the needs. The SCCA model therefore expanded to include members of the general pediatric anesthesia team (GA) with an interest in caring for children with CHD. To support safe, high‐quality care, SCCA practitioners and general pediatric anesthesiologists with cases that required pre‐assignment were scheduled in advance to allow proper preparation.

A structured system was developed collaboratively by the pediatric anesthesia and cardiac anesthesia (CA) divisions to determine the most appropriate team to safely care for children with CHD (Table 1).

**TABLE 1:** Assignment of CHD physiologies to anesthesia teams.

Recommendation	Congenital heart disease	Heart failure/cardiomyopathy	Valvar disease	Pulmonary hypertension	Rhythm disturbances
CA	On prostaglandin (PGE) due to ductal‐dependent pulmonary or systemic blood flow Not applicable for PGE for pulmonary hypertensive crisis	Extracorporeal support: Cardiopulmonary Bypass Cardiac ECMO VAD or other mechanical assist device	Significant outflow obstruction (including of mBTT shunt, Sano, or other outflow path) making extracorporeal support reasonably likely	Supra‐systemic RVP estimate with RV failure and high risk of requiring extracorporeal support	Unstable dysrhythmias with high risk of requiring extracorporeal support
GA SCCA pre‐assignment (Only applicable to 4th Floor Main OR)	Cyanotic CHD (palliated) Single ventricle physiology Unbalanced two ventricle physiology Williams syndrome (symptomatic stenosis) Severe pulmonary vein stenosis	Severe ventricular dysfunction ○ LV or RV ○ Systolic or Diastolic Heart transplant ○ Before 1st cath ○ Moderate to severe dysfunction Moderate to severe systolic anterior motion (SAM or HOCM) physiology	Severe RVOT obstruction: Subvalvar, Valvar, or Supravalvar Severe LVOT obstruction: Subvalvar, Valvar, or Supravalvar Severe valvular stenosis:	Severe pulmonary hypertension: Suprasystemic RV/PA pressure, regardless of therapy required	Unstable dysrhythmias: Symptomatic Brugada syndrome Pacemaker without underlying escape rhythm AICD with frequent cardioversion/defibrillation
GA with pre‐assignment	Patients with significant CHD with noncyanotic, 2‐ventricle repair, including: Balanced two ventricle physiology Williams syndrome patients without stenosis (or with asymptomatic stenosis) Pulmonary vein stenosis	Vascular bypass case Friedreich's Ataxia patients Moderate LV or RV ventricular dysfunction Stable intracardiac thrombosis Pericardial effusion with Tamponade physiology	Moderate RVOT obstruction: Subvalvar, Valvar, or Supravalvar Moderate LVOT obstruction: Subvalvar, Valvar, or Supravalvar Moderate valvular stenosis Severe valvular regurgitation Well‐functioning mechanical valve	Moderate pulmonary hypertension: RV pressure half systemic to systemic pressure, regardless of therapy required	Stable dysrhythmias: Pacemaker with documented escape rhythm AICD without need for frequent defibrillation
GA without pre‐assignment	Fully repaired cyanotic CHD w/2 ventricle physiology (e.g., ToF, TGA s/p arterial switch or congenitally corrected, TAPVR) Asymptomatic unrepaired CHD (e.g., ASD/VSD)	Mild LV/RV dysfunction Heart transplant After 1st cath No coronary disease Mild or no dysfunction Congenital Diaphragmatic Hernia (CDH) repair regardless medications or RV pressures VOGM (Vein of Galen Malformation) procedure regardless medications or RV pressures Lymphatic disease without cardiac disease	Any degree of PI or tricuspid regurgitation Mild valvular stenosis Moderate valvular regurgitation	Mild pulmonary hypertension RV pressure<half systemic	Atrial or ventricular ectopy well treated with antiarrhythmics or asymptomatic (e.g., isolated long QT syndrome, asymptomatic Brugada syndrome, PACs/PVCs)

Recognizing that many children with CHD can be managed by pediatric anesthesiologists, triaging and pre‐assignment were formalized into an organized process. When a procedural team wishes to schedule a child with CHD for a non‐cardiac procedure, a “Cardiac Anesthesia Evaluation” (CAE) is submitted to an EPIC in‐basket for review by a member of the cardiac anesthesia team, most often a nurse practitioner with extensive experience in this population. Each CAE is reviewed and assigned to one of four categories: Cardiac Anesthesia (CA) ~10% of cases, SCCA 15%, General Anesthesia (GA) with pre‐assignment 40%, or GA without pre‐assignment 40%. Approximately 2000 CAEs are submitted annually. As this team expanded, members identified a critical need for accessible, structured education in CHD physiology and anesthetic management. This recognition prompted the development of the *Cardiac Basics* curriculum, designed to build foundational cardiac knowledge across a broader provider base and to strengthen the workforce's capacity to safely meet patient needs.

## 2 Cardiac Basics: A Workforce Solution

### 2.1 Goals and Guiding Principles

The curriculum was developed with three primary goals:

1. To provide foundational cardiac knowledge across key CHD pathophysiologies.
2. To deliver content in a format accessible to a wide range of learners (anesthesiologists, fellows, CRNAs, intensivists, and trainees).
3. To maintain a psychologically safe learning environment that encourages open questioning and discussion.

### 2.2 Structure and Delivery

A comprehensive list of core topics (Table 2) was created, with flexibility for future expansion. Each week, a topic was presented across three platforms:

- Topic Guide: A 3–5‐page summary with objectives and references, distributed the Friday before.
- Interactive Didactics: Two identical 20‐min sessions (15‐min presentation +5 min for questions) offered on Monday and Wednesday at midday to maximize attendance without extending work hours. Sessions were available in person and virtually, with CME credit offered.
- Case‐Based Reinforcement: Clinical teams were encouraged to discuss the weekly topic during anesthetic care, allowing learners to hear diverse approaches and management strategies.

**TABLE 2:** Topics in the cardiac basics curriculum.

Category	Topics
General topics	Embryology
	Cardiopulmonary bypass
	Cardiac lesions, including surgical and catheter‐based interventions and anesthetic implications
Cardiac lesions	Atrial septal defect (ASD)
	Ventricular septal defect (VSD)
	Patent ductus arteriosus (PDA)
	Transposition of the great arteries (D‐TGA)
	Congenitally corrected TGA (L‐TGA)
	Tetralogy of fallot (TOF)
	Total anomalous pulmonary venous return (TAPVR)
	Truncus arteriosus (TA)
	Hypoplastic left heart syndrome/single ventricle physiology Atrioventricular canal defect (AVC)
	Interrupted aortic arch (IAA)
	Coarctation of the aorta
	Aortic stenosis
	Pulmonary stenosis
	Vascular rings
Syndromes	Trisomy 21
	22q11 deletion
	Noonan syndrome
	Turner syndrome
	Williams syndrome
	CHARGE syndrome
	VACTERL association
	Cardiomyopathies including Duchenne Muscular Dystrophy
Physiology	Basic cardiac equations
	Pulmonary artery (PA) bands
	Systemic‐to‐pulmonary artery shunts
	Fetal transition to adult circulation
Heart failure/interventions	Extracorporeal membrane oxygenation (ECMO)
	Ventricular assist devices (VADs)
	Heart transplantation
Anesthetic management	Electrophysiology studies
	Long QT syndrome
	Pulmonary hypertension
	Lymphatic interventions
	Principals of anesthetic management for the cath lab
	Anesthetic effects on the cardiovascular system
Miscellaneous	Near‐infrared spectroscopy (NIRS)
	Neonatal/infant tracheal intubation
	Umbilical arterial and venous catheter placement
	Methadone for pediatric cardiac surgery
	Indications for irradiation of blood products
	Pacemaker terminology
	Inhalational induction in CHD
	Infectious diseases—endocarditis, Kawasaki, rheumatic heart disease

The daily procedural schedule highlighted the weekly topic and upcoming relevant cases. A dedicated program coordinator ensures operational continuity by scheduling topics and speakers, distributing guides, arranging CME credit, and collecting feedback via QR‐coded surveys. Flexibility in scheduling was critical to sustaining faculty engagement.

### 2.3 Outcomes and Lessons Learned

In the first 35 weeks, more than 400 instances of attendance were recorded across all learner groups. Feedback was overwhelmingly positive: learners valued the brevity, clarity, and clinical applicability of the content. A total of 277 unique “quick evaluations” over the course of the first 18 months of the curriculum have been completed. The tool asked one question and had an open text box for comments. In response to “Please rate your level of agreement with this statement: This session was effective and useful to me” 93% of the respondents stated that they “strongly agree” with that statement and less than 1% of all other respondents stated they agree, were neutral, disagree or strongly disagree. Visual aids and case‐based discussion were particularly well received. Some learners noted that occasional presentations drifted into advanced content, underscoring the need to preserve psychological safety by keeping the focus on fundamentals. Faculty also benefited through scholarly credit for developing new educational materials.

### 2.4 Future Directions

Next steps include expanding the curriculum to additional participants while maintaining an inclusive environment, inviting senior cardiac CRNAs to develop and deliver sessions, and providing structured feedback to faculty on teaching effectiveness. Many topics will be repeated annually as cohorts change, and new topics will be added as needs evolve. The list of topics and topic guides has been expanded to broaden the coverage of topics on the American Board of Anesthesiology content outline for the Pediatric Anesthesiology board exam in recognition of the importance of this curricular piece for the pediatric anesthesia fellows. Additionally, we will continue to monitor the faculty's perception of the pediatric anesthesia fellows' readiness to care for this growing population of patients as the duration of this curriculum lengthens. Reinforcing the use of weekly guides during perioperative care will further extend the curriculum's impact. A survey has been developed to assess the pediatric anesthesiologists' comfort with caring for each of the categories of CHD patients to help determine impact and future directions of the series.

### 2.5 Broader Implications

*Cardiac Basics* represents a pragmatic and scalable response to the pediatric cardiac anesthesia workforce crisis. By equipping anesthesiologists and other providers with foundational cardiac knowledge, this curriculum extends the capacity of the workforce to care safely for selected CHD patients (Table 1). A complementary, and potentially more far‐reaching, strategy involves updating anesthesia residency and pediatric and adult cardiac anesthesia fellowship curricula to incorporate dedicated didactics and increase clinical exposure to CHD patients, ensuring that graduates are prepared to safely manage a subset of these cases. While pipeline repair, fellowship recruitment, and cultural change remain essential long‐term strategies, educational innovations like *Cardiac Basics* can provide an immediate, sustainable “force multiplier” for meeting patient needs.

## 3 Conclusion

With nearly one‐third of practicing pediatric cardiac anesthesiologists projected to retire or leave the subspecialty in the coming decade, and fellowship recruitment at historic lows, optimal anesthesia care for children with CHD is in jeopardy [4, 7]. Advances in medical and surgical care have ensured that most children born with CHD now survive into adulthood [1], but this success has generated unprecedented demand for anesthesia services. and expanded care outside the OR has further strained the workforce [2, 3].

Addressing this crisis requires long‐term strategies such as rebuilding the fellowship pipeline, aligning financial incentives and improving the professional culture [2, 7]. However, these efforts will take years to bear fruit. In the interim, pragmatic educational innovations are urgently needed to extend the capacity of the existing workforce.

The *Cardiac Basics* curriculum offers one such solution. By equipping anesthesiologists, fellows, CRNAs, and intensivists with the tools to safely manage defined subsets of CHD patients (Table 1), *Cardiac Basics* functions as a “force multiplier” that enhances workforce flexibility while preserving safety. The basics curriculum builds bridges between the groups caring for patients with CHD, ensuring that both the SCCA and general anesthesia faculty have clear access to expert consultation when needed. We propose that curricula modeled after *Cardiac Basics* can be readily adapted by other institutions to address similar workforce pressures.

## Data Availability

The data that support the findings of this study are available from the corresponding author upon reasonable request.
